# Pleural cytokines MIF and MIP-3α as novel biomarkers for complicated parapneumonic effusions and empyema

**DOI:** 10.1038/s41598-021-81053-6

**Published:** 2021-01-19

**Authors:** Chia-Yu Yang, Yu-Hsuan Kuo, Min Chen, Chih-Liang Wang, Li-Jane Shih, Yu-Ching Liu, Pei-Chun Hsueh, Yi-Hsuan Lai, Chi-Ming Chu, Chih-Ching Wu, Kuo-An Wu

**Affiliations:** 1 Department of Microbiology and Immunology, College of Medicine, Chang Gung University, Taoyuan, Taiwan; 2 Department of Otolaryngology-Head and Neck Surgery, Chang Gung Memorial Hospital, Taoyuan, Taiwan; 3 Molecular Medicine Research Center, Chang Gung University, Taoyuan, Taiwan; 4 Graduate Institute of Biomedical Sciences, College of Medicine, Chang Gung University, Taoyuan, Taiwan; 5 School of Medicine, College of Medicine, Chang Gung University, Taoyuan, Taiwan; 6 Division of Pulmonary Oncology and Interventional Bronchoscopy, Department of Thoracic Medicine, Chang Gung Memorial Hospital, Taoyuan, Taiwan; 7 Department of Medical Laboratory, Taoyuan Armed Forces General Hospital, Taoyuan, Taiwan; 8 Division of Biomedical Statistics and Informatics, School of Public Health, National Defense Medical Center, Taipei, Taiwan; 9 Department of Medical Biotechnology and Laboratory Science, College of Medicine, Chang Gung University, Taoyuan, Taiwan; 10 Department of Internal Medicine, Taoyuan Armed Forces General Hospital, Taoyuan, Taiwan; 11 School of Medicine, Fu Jen Catholic University, New Taipei City, Taiwan

**Keywords:** Biomarkers, Translational research, Cytokines

## Abstract

Patients with complicated parapneumonic effusion (CPPE)/empyema have high morbidity and mortality, particularly when adequate management is delayed. We aimed to investigate novel dysregulated cytokines that can be used as biomarkers for infectious pleural effusions, especially for CPPE/empyema. Expression of 40 cytokines in parapneumonic effusions (PPE) was screened in the discovery phase, involving 63 patients, using a multiplex immunobead-based assay. Six cytokines were subsequently validated by enzyme-linked immunosorbent assays (ELISAs). We then used ELISA to further evaluate the diagnostic values and cutoff values of these cytokines as potential biomarkers in an expanded group that included 200 patients with uncomplicated parapneumonic effusion (UPPE), CPPE, empyema, transudates, other exudates, and malignant pleural effusion (MPE). The pleural levels of four cytokines (MIF, MIP-3α, IL-1β, ENA-78) were highest and significantly increased in CPPE/empyema compared with those in other etiologies. According to receiver operating characteristic curve analysis, the four cytokines (MIF, MIP-3α, IL-1β, and ENA-78) had areas under the curve (AUCs) greater than 0.710 for discriminating parapneumonic pleural effusion from noninfectious pleural effusions. In a comparison of nonpurulent CPPE with UPPE, logistic regression analysis revealed that pleural fluid MIF ≥ 12 ng/ml and MIP-3α ≥ 4.3 ng/ml had the best diagnostic value; MIF also displayed the highest odds ratio of 663 for nonpurulent CPPE, with 97.5% specificity, 94.44% sensitivity, and an AUC of 0.950. In conclusion, our results show that elevated MIF and MIP-3α may be used as novel biomarkers for PPE diagnosis, particularly in patients with CPPE/empyema; the findings indicate that dysregulated cytokine expression may provide clues about the pathogenesis of pleural infection.

## Introduction

Pleural effusion results from the accumulation of excess fluid in the pleural cavity; it can be classified as transudate or exudate based on clinical characterization^1,2^. The most common causes of exudates are pneumonia, lung abscess, and malignancy^3^. Approximately 20–40% of patients who are hospitalized for pneumonia develop parapneumonic effusion (PPE)^4^. PPE is the fluid that accompanies acute bacterial pneumonia or other infections of the lung parenchyma; it can be classified into three stages based on disease progression and pathophysiology: uncomplicated PPE (UPPE), complicated PPE (CPPE), and thoracic empyema^4–6^. The progression of PPEs to CPPE/empyema is associated with poor clinical outcome and increased mortality^5,7^. Most patients with UPPE resolve with appropriate antibiotic therapy, and drainage is generally not necessary. In most cases of CPPE/empyema, patients require pleural drainage with or without fibrinolytics or thoracic surgery^8^. In addition, CPPE/empyema also has a poorer prognosis than UPPE, particularly when adequate management is delayed^9^. Traditionally, the criteria for clinical diagnosis of CPPE based on pleural fluid are a lactate dehydrogenase (LDH) level above 1000 U/l, a glucose level below 60 mg/dl, or a pH below 7.20; empyema refers to a condition in which there is pus in the pleural space^3–5^.

Inflammation of the pleura results in increased pleural fluid accumulation and neutrophil infiltration of the pleural cavity^10^. This pleural fluid contains various inflammatory mediators, cytokines, and metabolites^11,12^. Cytokines are secreted proteins that are involved in many inflammatory responses and regulate the migration of immune cells in response to various stimuli^13^. They bind to specific receptors on their target cells, leading to activation of the target cells and amplification of immune responses^13^. The pleural levels of some cytokines in patients with different etiologies have been reported previously, and some of the cytokines that show elevated levels in pleural effusions have been proposed as biomarkers for differential diagnosis of pleural effusion. Increased levels of IL-6, IL-8, and IL-1β have been reported in infectious pleural effusion compared with malignant pleural effusion and transudates^14^. Among the PPEs, IL-8 and IL-1β levels were found to be much higher in CPPE than in UPPE^15,16^. Porcel et al. reported that TNF-α in pleural fluid is a marker of CPPE^12^. However, large-scale screening of cytokines in individual patients with pleural effusion is still lacking. In the present study, we aimed to identify novel dysregulated cytokines to serve as biomarkers for infectious pleural effusion, particularly patients with CPPE/empyema. To lower down the sample usage, forty cytokines in parapneumonic pleural effusions were quickly screened using a multiplex immunobead-based system. The potential dysregulated cytokines were further validated using sandwich ELISA, which is easily used in clinical practice for translational medicine, in the expanded group that included six different types of pleural effusions. We then used receiver operating characteristic (ROC) curves and logistic regression analysis to evaluate the potential utility of these cytokines as biomarkers for the diagnosis of CPPE/empyema.

## Results

### Profile of 40 cytokines in pleural effusions from PPE patients

The patients’ demographic data and the pleural levels of the biochemical parameters are shown in Table 1. In the first cohort, we screened the pleural levels of 40 cytokines in 63 patients with PPE (empyema: 20 patients; nonpurulent CPPE: 13 patients; UPPE: 30 patients) using a Bio-Plex Pro Human Chemokine multiplex immunoassay. The 40 candidate proteins (10 cytokines, 27 chemokines, 2 growth factors, and 1 other) were selected for screening because these proteins have been reported to regulate inflammation and immune cell function/migration in gene ontological analyses (Supplementary Table S1). Among the 40 cytokines we screened as the following strategy because of the limited samples (Table 2); (1) the fold changes of fluorescence intensities were upper 1.3 and lower 0.76 between CPPE and UPPE, (2) ten cytokines (ENA-78, IL-1β, Groβ, IL-8, MIP-3α, MIF, fractalkine, MCP3, MIP-1α, and Groα) were increased, (3) two cytokines (CTACK and SDF1) were decreased, (4) twelve cytokines were selected and examined the statistical significance between CPPE and UPPE using Mann–Whitney U test, (5) Bonferroni-adjusted p values < 0.05/24 (12 cytokines and 2 comparisons) = 0.0021 indicated significance. The results showed that 9 up-regulated cytokines and 1 down-regulated cytokine showed statistical significantly difference (Table 2).

**Table 1:** Clinical characteristics of the patients in this study.

Characteristics	Empyema	CPPE	UPPE	*p* value^b^	*p* value^c^			
**Discovery phase for multiplex immunobead-based assay**								
Patients	20	13	30	–	–			
Male (%)	17 (85.0%)	12 (92.3%)	26 (86.7%)	–	–			
Age (years) ^a^	67.80 ± 4.07	65.15 ± 4.55	67.33 ± 3.46	0.863	0.758			
Protein (g/dl) ^a^	3.85 ± 0.32	4.39 ± 0.23	3.64 ± 0.16	0.023	0.573			
Glucose (mg/dl) ^a^	45.72 ± 12.46	41.05 ± 14.02	158.22 ± 14.98	< 0.001	0.235			
LDH (U/l) ^a^	8066 ± 2751	2267 ± 672	425 ± 55	< 0.001	0.013			
pH^a^	6.89 ± 0.08	7.19 ± 0.03	7.43 ± 0.10	< 0.001	0.002			

Characteristics	Empyema	CPPE	UPPE	Other exudates	Transudates	MPE	*p* value^b^	*p* value^c^
**Six types of pleural effusion for ELISAs**								
Patients	22	18	40	31	40	49	–	–
Male (%)	19 (86.3%)	15 (83.3%)	31 (77.5%)	21 (67.7%)	25 (62.5%)	24 (48.9%)	–	–
Age (years) ^a^	67.82 ± 3.95	60.22 ± 4.11	68.18 ± 3.14	76.09 ± 3.22	74.37 ± 2.65	67.16 ± 2.54	0.143	0.17
Protein (g/dl) ^a^	3.90 ± 0.28	4.62 ± 0.20	3.59 ± 0.15	3.66 ± 0.21	1.81 ± 0.10	4.02 ± 0.17^d^	< 0.001	0.075
Glucose (mg/dl) ^a^	38.68 ± 10.58	49.26 ± 12.64	158.83 ± 11.67	140.64 ± 9.70	158.98 ± 7.97	123.28 ± 7.42^d^	< 0.001	0.178
LDH (U/l) ^a^	6126 ± 1926	1451 ± 297	406 ± 48	186 ± 20	83 ± 4	460 ± 50^d^	< 0.001	0.005
pH^a^	6.95 ± 0.07	7.19 ± 0.03	7.44 ± 0.02	7.44 ± 0.01	7.47 ± 0.01	NA	< 0.001	0.019

^a^Data are presented as the mean ± SEM. *NA* not available, *UPPE* uncomplicated parapneumonic effusion, *CPPE* nonpurulent complicated parapneumonic effusion, *MPE* malignant pleural effusion.

The *p* value on two-tailed Student’s *t* test indicates the difference between ^b^UPPE and CPPE or the ^c^empyema and CPPE. ^d^Protein, glucose, and LDH data are missing for two patients with MPE.

**Table 2:** Cytokine profiling in the discovery phase with the multiplex immunobead-based assay.

Cytokine^a^	Empyema (n = 20)		CPPE (n = 13)		UPPE (n = 30)		CPPE vs UPPE		Empyema vs CPPE	
	Mean	SEM	Mean	SEM	Mean	SEM	Fold change	p value^b^	Fold change	p value^c^
**Upregulated**										
ENA78	960.3	389.45	311.23	169	15.17	10.8	20.52	< 0.001*	3.09	0.11
IL-1β	4618.85	1098.71	1575.31	661.93	109.72	28.02	14.36	< 0.001*	2.93	0.03
Groβ	644.35	507.02	29.54	11.63	3.65	0.94	8.09	< 0.001*	21.81	0.11
IL-8	17,724.98	1929.63	11,784.88	2346.18	2084.88	734.5	5.65	< 0.001*	1.5	0.02
MIP-3α	8710.53	1400.22	9136.23	1933.32	1648.75	466.3	5.54	0.002*	0.95	1.00
MIF	7898.83	1281.88	5171.53	1405.81	944.45	406.91	5.48	< 0.001*	1.53	0.11
Fractalkine	1483.75	224.32	1512.99	311.15	286.28	68.39	5.28	< 0.001*	0.98	0.84
MCP3	57.1	22.33	24.42	4.07	7.5	1.9	3.26	< 0.001*	2.34	0.57
MIP-1α	2309.93	835.01	447.96	259.64	159.68	57.75	2.81	0.012	5.16	0.02
Groα	1038.58	791.26	118	31.68	84.9	49.86	1.39	0.002*	8.8	0.25
**Downregulated**										
CTACK	145.55	42	206	77.19	379.48	31.09	0.54	< 0.001*	0.71	0.194
SDF1	336.13	151.88	454.27	120.41	902.57	75.12	0.5	0.003	0.74	0.043

^a^Data are presented as the fluorescence intensity measured in a multiplex immunobead-based assay. *UPPE* uncomplicated parapneumonic effusion, *CPPE* nonpurulent complicated parapneumonic effusion.

Statistical analysis was done using Mann–Whitney U test for comparing the difference between ^b^CPPE and UPPE or ^c^Empyema and CPPE. Bonferroni-adjusted p-values < 0.05/24 (12 cytokines and 2 comparisons) = 0.0021 indicate significance.

### Validation of pleural cytokine levels in six types of pleural effusions

Because of a lack of standard proteins in this phase, only the relative abundance of the cytokines in each sample was assessed, as based on the fluorescence intensity acquired in the multiplex immunoassay. To validate the results in the multiplex immunoassay, the 6 cytokines (MIF, MIP-3α, IL-1β, ENA78, Groα, and Groβ) were further detected with the same PPE samples using sandwich ELISAs in which standard proteins were used to determine the absolute concentrations of the cytokines (Table 3). IL-1β has been reported to increase in CPPE/empyema, the pleural level of IL-1β was determined as a control^16^. However, because of an insufficient volume for 16 samples, only 47 samples (17 empyema, 8 CPPE, and 22 UPPE) were available and used to perform the ELISAs. The correlation of the 6 cytokines between the multiplex immunoassay and the sandwich ELISAs is shown in Supplementary Fig. S1. The cytokine levels between the 2 platforms showed a significantly positive association. The correlation coefficients between two platforms ranged from 0.69 to 0.87 (all *p* values < 0.0001; *r* = 0.694 for MIF, *r* = 0.890 for MIP-3α, *r* = 0.828 for IL-1β, *r* = 0.832 for ENA-78, *r* = 0.875 for Groβ, *r* = 0.624 for Groα) (Supplementary Fig. S1).

**Table 3:** The concentration of six cytokines in PPE patients using ELISA.

		Empyema (n = 22)		CPPE (n = 18)		UPPE (n = 40)		CPPE vs UPPE	
Cytokine^a^	Patient	From discovery phase n = 17	New patient n = 5	From discovery phase n = 8	New patient n = 10	From discovery phase n = 22	New patient n = 18	Discovery phase p value^b^	New patients p value^c^
MIF	Mean, median	76,637, 76,380	78,640, 50,619	45,963, 55,148	47,133, 41,518	3165, 2890	3406, 2401	0.001*	< 0.001*
	25% Percentile	33,450	34,334	22,165	27,863	331.7	186		
	75% Percentile	112,619	136,956	64,711	65,162	5378	4749		
MIP-3α	Mean, median	9086, 12,243	8500, 10,308	8190, 9820	8398, 9621	1080, 65	1295, 226	< 0.001*	< 0.001*
	25% Percentile	3094	4456	4919	6656	16.4	19.6		
	75% Percentile	14,341	11,641	11,730	10,177	2841	2004		
IL-1β	Mean, median	733, 644	761, 734	309, 264	350, 251	33.9, 5.5	56.7, 4.2	0.006	< 0.001*
	25% Percentile	327	408	107	63.1	3.8	4.6		
	75% Percentile	1064	1128	381	645	38.5	71.5		
ENA78	Mean, median	17,277, 5535	14,900, 18,637	18,992, 12,245	15,656, 8189	161, 16.8	368, 35.6	< 0.001*	< 0.001*
	25% Percentile	369	2539	346	446	15.3	16.5		
	75% Percentile	33,570	25,393	36,802	28,974	18.8	516		
Groβ	Mean, median	432, 75	344, 168	495, 150	305, 125	37.2, 18.6	53.2, 28.2	0.005	0.004
	25% Percentile	15.6	52.4	38.6	42.7	16.9	15.2		
	75% Percentile	663	724	289	427	20.1	41		
Groα	Mean, median	1096, 250	1185, 484	1520, 714	1038, 653	236, 48	252, 91.2	0.063	0.308
	25% Percentile	31.2	138	33.8	33.9	33.6	43.2		
	75% Percentile	1675	2581	2642	1819	317	450		

^a^Data are presented as mean, median, and 25th–75th percentile (pg/ml). UPPE: uncomplicated parapneumonic effusion; CPPE: nonpurulent complicated parapneumonic effusion. Statistical analysis between CPPE and UPPE was done using Mann–Whitney U test. Bonferroni-adjusted p values < 0.05/12 (6 cytokines and 2 comparison) = 0.004 indicate significance.

**Figure 1:**
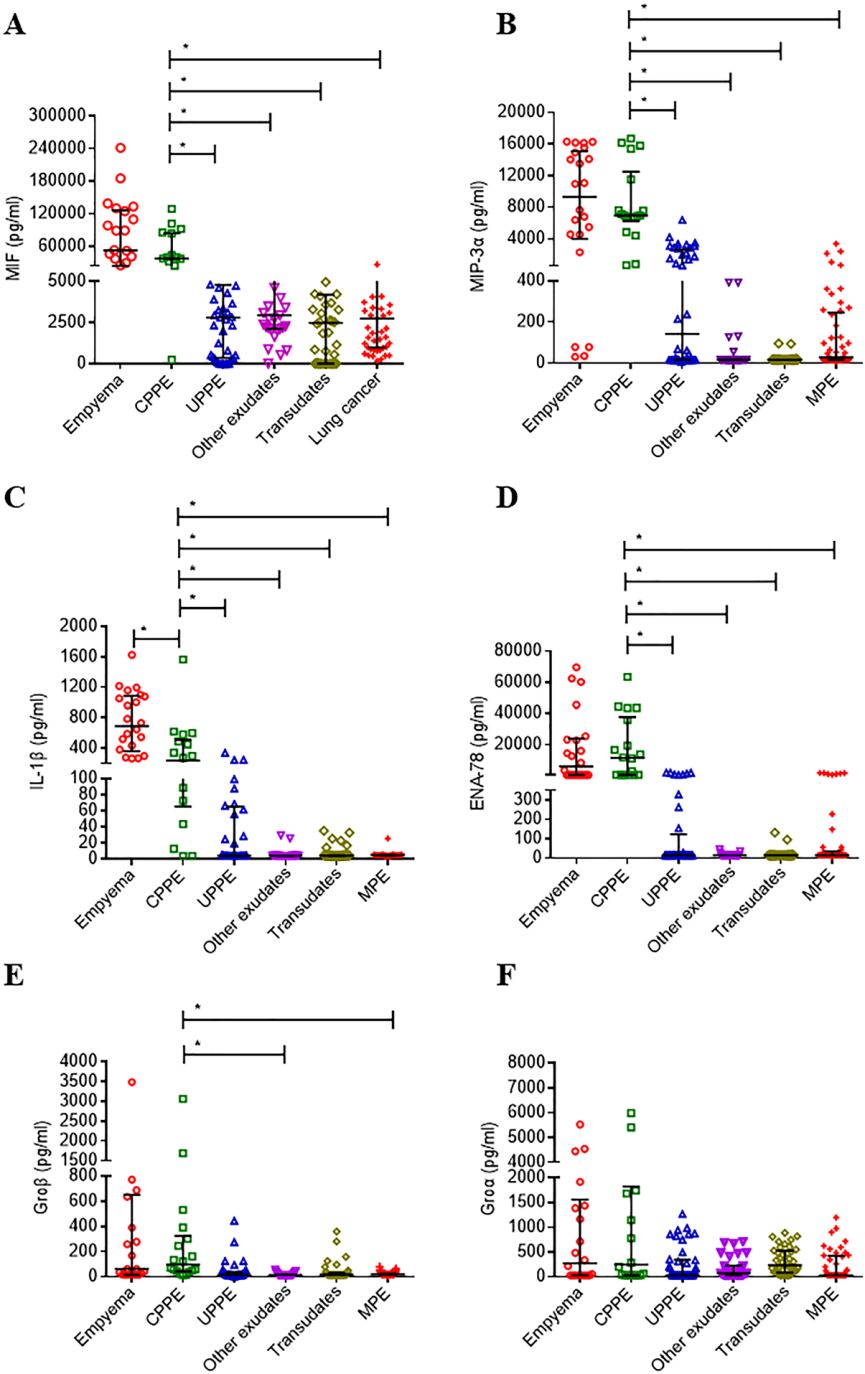
Pleural levels of six dysregulated cytokines in various types of pleural effusion. The pleural levels of MIF (**A**), MIP-3α (**B**), IL-1β (**C**), ENA-78 (**D**), Groβ (**E**), and Groα (**F**) were measured by ELISA in patients with empyema, complicated parapneumonic effusion (CPPE), uncomplicated parapneumonic effusion (UPPE), other exudates, transudates, and malignant pleural effusion (MPE). The middle lines represent median values. The interval lines represent the 25th and 75th percentile. Statistical analysis was done using the post hoc Tukey test. Bonferroni-adjusted p values < 0.05/30 (6 cytokines and 5 comparisons) = 0.00166 indicate significance.

Based on the ELISA results, the levels of three cytokines (MIF, MIP-3α, ENA78) in CPPE samples were significantly higher than in UPPE samples from the discovery phase (p < 0.004) (Table 3). We therefore investigated cytokine levels in the expanded 33 PPE samples (5 empyema, 10 CPPE, and 18 UPPE) by ELISA. Using the new 33 PPE samples, we confirmed that MIF, MIP-3α, and ENA78 were increased in CPPE samples compared with UPPE samples (Table 3). There was a consistent trend for increased IL-1β in CPPE compared to UPPE in two populations, but these differences did not reach statistical significance in the discovery phase. This may be caused by limited samples. Furthermore, we combined the data for the 80 PPE patients (Table 3) and 120 pleural effusion samples (49 MPE, 40 transudates, and 31 exudates) to evaluate the diagnostic potentials and cutoff values of the 5 cytokines for CPPE/empyema detection (Table 4). Among the six different types of effusions, the mean concentrations of MIF, MIP-3α, IL-1β, and ENA-78 were highest and most significantly increased in CPPE/empyema relative to those in effusions of other etiologies, including UPPE, other exudates, transudates, and MPE (Fig. 1; Table 4). In addition, the pleural levels of these proteins in PPE, including CPPE/empyema and UPPE, were significantly higher than those in noninfectious PE. Statistical analysis was done using the post hoc Turkey test for comparison the six cytokines among six different subgroups of patients. When we compared the pleural levels of these cytokines in CPPE with the levels in UPPE, we found that the pleural levels of these proteins (MIF, MIP-3α, IL-1β, and ENA-78) were significantly increased in CPPE compared to UPPE (Table 4). Interesting, the pleural levels of MIF and IL-1β were significantly higher in empyema compared with CPPE (Table 4).

**Table 4:** Pleural effusion levels of six cytokines in six types of pleural effusion by ELISAs.

Cytokine^a^		Empyema (n = 22)	CPPE (n = 18)	UPPE (n = 40)	Other exudates (n = 31)	Transudates (n = 40)	MPE (n = 49)	p value^b^	p value^c^
MIF	Mean, median	77,093, 52,553	46,612, 37,269	3273, 2813	6041, 2947	3145, 2480	5210, 2750	1.04E−03*	1.35 E−08*
	25th–75th percentile	24,276–126,314	21,799–84,219	358–4783	2122–10,030	12.1–4188	986.6–7192		
MIP-3α	Mean, median	8953, 9294	8305, 6922	1177, 141.7	48.1, 15.9	19.6, 16.0	271.4, 27.8	9.70 E−01	5.28 E−13*
	25th–75th percentile	3978–15,086	6237–12,481	15.6–2637	15.7–16.0	15.8–16.2	15.6–245.7		
IL-1β	Mean, median	740.1, 689.6	332.1, 235.2	44.2, 4.2	5.33, 4.1	6.95, 4.4	4.24, 4.3	1.06 E−10*	4.55 E−07*
	25th–75th percentile	357.9–1084	65.3–511.2	4.0–65.0	3.9–4.3	4.1–4.7	4.2–5.3		
ENA78	Mean, median	16,737, 5923	17,139, 11,368	254.4, 18.1	17.6, 20.2	20.6, 18.9	206.8, 23.1	1.00E+00	3.90 E−08*
	25th–75th percentile	390.4–23,833	400.5–37,684	16.4–122.9	18.1–20.9	17.9–19.8	22.3–36.3		
Groβ	Mean, median	412, 61.8	389, 97	44.4, 15.8	20.1, 15.8	43.3, 16.3	23.7, 20	1.00E+00	6.74 E−03
	25th–75th percentile	15.8–650.2	42.3–325	15.7–36.2	15.7–20.7	15.8–31.8	17.4–26.8		
Groα	Mean, median	1116, 278	1252, 245	243.5, 31.8	185, 73	318, 230	273, 32.1	9.96 E−01	5.53 E−03
	25th–75th percentile	31.4–1555	31.3–1819	31.2–346.2	31.6–225	87.3–526	31.7–421.9		

^a^Data are presented as mean, median, and 25th–75th percentile (pg/ml). *UPPE* uncomplicated parapneumonic effusion, *CPPE* nonpurulent complicated parapneumonic effusion, *MPE* malignant pleural effusion.

Statistical analysis was done using the post hoc Tukey test. Bonferroni-adjusted p-values < 0.05/30 (6 cytokines and 5 comparisons) = 0.00166 indicate significance between ^b^empyema vs CPPE or ^c^CPPE vs UPPE.

### Diagnostic values of these cytokines for distinguishing PPE from noninfectious PE

We then characterized the diagnostic value of these markers for distinguishing PPE (empyema, CPPE and UPPE) from noninfectious PE (other exudates, transudates, and MPE). The true positive rate (sensitivity) was plotted against the false positive rate (100%-specificity), and the area under the curve (AUC) was reported with a 95% confidence interval as an estimate of diagnostic usefulness. Two markers (MIP-3α and IL-1β) had AUCs higher than 0.820 for distinguishing PPE from noninfectious PE (0.828 for MIP-3α; 0.820 for IL-1β) (Table 5).

**Table 5:** Diagnostic accuracy of six cytokines for distinguishing between PPE and noninfectious PE.

Cytokine	AUC (95% CI)	Cutoff	Specificity (%)	Sensitivity (%)	PPV (%)	NPV (%)
MIF (pg/ml)	0.710 (0.630–0.789)	> 4800	73.33	61.25	60.49	73.95
MIP-3α (pg/ml)	0.828 (0.762–0.895)	> 450	95.83	67.50	91.52	81.56
IL-1β (pg/ml)	0.820 (0.753–0.888)	> 40	99.17	61.25	98.00	79.47
ENA78 (pg/ml)	0.771 (0.699–0.843)	> 150	93.33	62.50	86.20	78.87
Groβ (pg/ml)	0.670 (0.588–0.752)	> 30	77.50	51.25	60.29	70.46
Groα (pg/ml)	0.528 (0.441–0.615)	–	–	–	–	–

Parapneumonic effusion (PPE, refers to UPPE, CPPE, and empyema) and noninfectious PE (refers to other exudates, transudates, and MPE) in this study.

*PPV* positive predictive value, *NPV* negative predictive value, *AUC* area under ROC curve.

### Diagnostic values of these cytokines for distinguishing CPPE from UPPE

CPPE/empyema generally has a poorer prognosis than other types of effusion, particularly when immediate medical management is delayed. To determine the efficacies of the six tested cytokines as potential markers for distinguishing nonpurulent CPPE from UPPE, logistic regression and receiver operating characteristic curve analysis were performed. Among the potential markers, MIF and MIP-3α had AUCs higher than 0.950 for distinguishing CPPE from UPPE (Table 6, Supplementary Fig. S2). Univariate logistic regression analysis demonstrated a strong association between MIF levels > 12 ng/ml and CPPE, with a high odds ratio of 663, a specificity of 97.50%, and a sensitivity of 94.44% for distinguishing CPPE from UPPE (Table 6). Furthermore, MIP-3α had an odds ratio of 312, a specificity of 95.12%, and a sensitivity of 94.11% for distinguishing CPPE from UPPE (Table 6). The Spearman correlations among the pleural fluid levels of the six proteins and clinical parameters (glucose, LDH, and pH) in all PPE patients were calculated. The levels of the six proteins in pleural effusions showed a statistically significant positive correlation (*p* < 0.05; Supplementary Table S2). In conclusion, our results identify elevated MIF and MIP-3α as novel biomarkers for PPE diagnosis, particularly in patients with CPPE/empyema.

**Table 6:** Diagnostic accuracy of six cytokines for distinguishing between CPPE and UPPE.

Cytokine	AUC (95% CI)	Cutoff	Specificity (%)	Sensitivity (%)	PPV (%)	NPV (%)	Odds Ratio (95% CI)
MIF (pg/ml)	0.950 (0.862–1.000)	> 12,000	97.50	94.44	94.44	97.50	663.00 (39.13–11,231.83)
MIP-3α (pg/ml)	0.953 (0.891–1.000)	> 4300	95.12	94.11	88.88	97.50	312.00 (26.39–2688.51)
IL-1β (pg/ml)	0.847 (0.730–0.964	> 145	86.04	80.00	66.66	92.50	24.66 (5.33–114.05)
ENA78 (pg/ml)	0.932 (0.870–0.994)	> 400	82.50	61.11	61.11	82.50	7.40 (2.12–25.86)
Groβ (pg/ml)	0.840 (0.727–0.954	> 60	83.33	68.75	61.11	87.50	11.00 (2.90–41.70)
Groα (pg/ml)	0.671 (0.509–0.833)	–	–	–	–	–	–

*PPV* positive predictive values, *NPV* negative predictive values, *AUC* area under ROC curve.

## Discussion

In the discovery phase of this study, we conducted comprehensive cytokine screening of pleural fluid specimens from PPE patients using a multiplex immunoassay. Among the 40 screened cytokines, we identified two, IL-1β and IL-8, that were present at elevated levels and have previously been reported to increase in CPPE/empyema compared to UPPE^15^. We further identified seven additional cytokines (MIF, MIP-3α, ENA-78, Groβ, Groα, MCP3, and fractalkine) that have not been reported to increase significantly in CPPE/empyema compared to UPPE. The pleural levels of CTACK have not been reported to decrease significantly in CPPE/empyema compared to UPPE. Therefore, the multiplex immunobead-based assay not only assisted in confirming the reported changes in cytokine levels that occur in PPE but also served as a tool for the discovery of novel dysregulated cytokines in PPE.

The possibility that increased pleural levels of MIF and MIP-3α may serve as biomarkers for distinguishing PPE, especially CPPE/empyema, from noninfectious PE was first explored in this study. MIF is an important regulator of protective responses to intracellular pathogens^17,18^; it is also linked to the pathogenesis of several inflammatory disorders^19–22^. High concentrations of MIF have been found in the plasma of patients with severe sepsis^21–23^. MIF is increased in the blood and bronchoalveolar lavage of patients with acute respiratory distress syndrome and is associated with disease outcome^24,25^. However, pleural levels of MIF have never been reported in PPE. In patients with parapneumonic effusion, pleural MIF may be released by macrophages, lymphocytes, and pulmonary epithelial cells in response to microbial stimuli, and MIF may contribute to the chemoattraction of neutrophils to the pleural cavity and enhance the production of other cytokines in PPE. Recently, Lang et al. reported that MIF may participate in the activation of the NLRP3 inflammasome^26^. IL-1β is a proinflammatory cytokine in the toll-like receptor signaling and NLRP3 inflammasome pathways, which are crucial for host defense responses to infection. IL-1β levels were also significantly increased in PPE, especially in CPPE/empyema, and had an AUC of 0.847 for identifying nonpurulent CPPE from UPPE in our cohort; this finding is consistent with a previous report. The roles of MIF and NLRP3 inflammasome-induced IL-1β in PPE may need further investigation.

In this report, we first identified other two chemokines, Groα/CXCL1 and Groβ/CXCL2, that are also elevated in PPE, especially in CPPE/empyema, but these differences did not reach statistical significance. The diagnostic power of these chemokines was not sufficient for clinical diagnosis. Paudel et al*.* reported that Groα/CXCL1 is a central player in host defense, granulopoiesis, and mobilization of neutrophils during bacterial pneumonia-induced sepsis in a murine model^27^. Filippo et al*.* also reported that Groα/CXCL1 and Groβ/CXCL2 control neutrophil recruitment in a murine model^28^. The pleural levels of Groα/CXCL1 and Groβ/CXCL2 in pneumonia patients have not been investigated previously; the role of these two cytokines may need further study. MIP-3α/CCL20 is involved in the chemoattraction of immature dendritic cells and effector/memory T- and B-cells to skin and mucosal surfaces^29^. It had been reported that patients with multiple sclerosis or ischemic heart disease have higher circulating levels of chemokine MIP-3α^30,31^. MIP-3α is upregulated in the cerebrospinal fluid (CSF) of patients with pneumococcal meningitis^32^.

A higher level of MIP-3α in PPE than in MPE was previously reported, but that report did not include patients with other types of pleural effusion^33^. We further identified that the pleural level of MIP-3α was higher in PPE, especially in CPPE/empyema, than in other types of pleural effusion including UPPE, transudates, and other exudates. ENA78 has been reported to regulate neutrophil homeostasis and recruitment. It has been reported that compared with noninfectious pleural effusion (19 with malignant pleural effusion, 21 with tuberculous pleural effusion, 17 with transudates), the pleural levels of ENA78 were significantly increased in infectious pleural effusion (n = 18)^34^. In our study, we further classified infectious pleural effusions as empyema, CPPE, or UPPE; ENA-78 levels were significantly increased in CPPE/empyema compared with UPPE.

Among the patients with PPE, the mechanism of progression from UPPE to CPPE/empyema is unclear. Findings from previous reports and our study of PPE suggest that elevated pleural levels of various inflammatory cytokines, including IL-6, IL-8, TNF-α, MIF, MIP3α, IL-1β, ENA78, Groα, and Groβ, may contribute to the development of pleural inflammation. The proinflammatory cytokines (IL-6, IL-8, TNF-α) initiate the immune activation cascade, leading to stimulation of immune cells and epithelial cells and the production of other cytokines. Elevated levels of MIF, ENA78, Groα, and Groβ may enhance the influx of neutrophils into the pleural space. In addition, MIF induces excessive macrophage and neutrophil activation, leading to IL-1β production. At the end stage, toxic neutrophil granule proteins are released, and fibroblast proliferation and fibrin deposition result in pus formation. Limited by our sample size, Groα and Groβ levels displayed an increasing trend in CPPE/empyema compared to other conditions, though a significant difference was not reached. Nevertheless, levels of Groα and Groβ in pleural effusions are an important issue, and further characterization is needed.

In addition to the above, we demonstrated that two cytokines, CTACK and SDF-1, are downregulated in CPPE/empyema compared to UPPE and potentially identified a previously unknown involvement of CTACK and SDF-1 in pneumonia or pleuritis. CTACK, also known as chemokine ligand 27 (CCL27), was reported to attract memory T cells for assistance with T cell-mediated inflammation in the skin^35^. Serum CTACK levels in patients with atopic dermatitis, psoriasis vulgaris, or alopecia areata are higher than those in healthy control subjects^36^. The role of CTACK in pneumonia has not been described. SDF-1, also known as C-X-C motif chemokine 12 (CXCL12), is ubiquitously expressed in many tissues and cell types. Bladder epithelial cells can secrete SDF-1, thereby initiating the accumulation of immune cells, including lymphocytes, monocytes, and neutrophils, at the site of infection during urinary tract infection^37^. Burgoyne et al. observed that SDF-1 levels in synovial tissues were increased in RA patients who experienced relapse compared with RA patients who experienced remission^38^. Tsai et al*.* observed that plasma levels of SDF-1 were elevated in patients with community-acquired pneumonia and were associated with disease severity^39^. The observed SDF-1 plasma levels in pneumonia patients were approximately 2–4 ng/ml^39^. Pleural levels of CTACK and SDF-1 need to be further validated by ELISA assay in the future. The roles of CTACK and SDF-1 in regulating T cell homeostasis and function in PPE also need to be characterized.

The study presented herein has some limitations. The first limitation is that we were unable to analyze tuberculous pleural effusion. The second limitation is the lack of a longitudinal cohort. This study is the first to report that various cytokines are dysregulated in individual PPE patients. The imbalance among cytokines at different disease stages may influence the outcomes of infectious diseases. Individuals who are at increased risk of morbidity might exhibit multiple dysregulated cytokines. Thus, longitudinal cohorts would be valuable for further study.

In conclusion, the present study reveals that the levels of various cytokines in pleural fluid are significantly associated with CPPE/empyema based on comprehensive cytokine screening. Our present findings not only identify novel upregulated cytokines that allow improved identification of CPPE/empyema but also reveal cytokine networks that contribute to our understanding of the potential immunopathological mechanisms involved in pleuritis.

## Methods

### Study design and population

In this prospective study, a total of 216 patients were enrolled from June 2014 to June 2018. The participants in the first cohort were enrolled at Taoyuan Armed Forces General Hospital, Taiwan. The participants in the ELISA platform were enrolled at Taoyuan Armed Forces General Hospital and Chang Gung Memorial Hospital, Taiwan. The study was approved by the Institutional Review Boards of the Tri-Service General Hospital (TSGH) and Chang Gung Memorial Hospital (CGMH), Taiwan (approval number: 105-2109C, 201700601B0, TY102-06). Patients with PPE, other exudates, and transudates were all enrolled in Taoyuan Armed Forces General Hospital. MPE is an advanced disease usually requiring surgical removal of cancer. Chang Gung Memorial Hospital Taiwan is a medical center at Taoyuan City responsible for cancer patients transferred from the Taoyuan Armed Forces General Hospital. Therefore, the samples of MPE were collected at Chang Gung Memorial Hospital. All experiments were performed in accordance with the guidelines and regulations set forth by the Institutional Review Boards of TSGH and CGMH. Prior to sample collection, written informed consent to participation in the study was obtained from all patients. All adult patients with pleural effusion who underwent diagnostic thoracentesis were considered for enrollment in this study by the attending physicians. If a patient underwent more than one thoracentesis during the hospitalization period, the samples obtained at the first tap were considered. The patients were divided into six groups according to the cause of the pleural effusion: transudates (40 patients), malignant pleural effusions (MPE; 49 patients), UPPE (48 patients), nonpurulent CPPE (23 patients), empyema (25 patients) and other exudates (31 patients). Transudates were effusions secondary to heart failure (22 patients), hypoalbuminemia (13 patients), liver cirrhosis (3 patients), or renal failure (2 patients). The primary tumors in malignant effusions were lung (40 patients), breast (1 patient), unknown (7 patients), and lymphoma (1 patient). Among the other exudates, there were 9 idiopathic, 7 trapped lungs, 4 intraabdominal infection, 2 postcardiac injury syndrome, 2 post-heart surgery, 2 pulmonary embolisms, 1 hemothorax, 1 uremic pleurisy, 1 Sjögren's syndrome, 1 aortic dissection, and 1 chylothroax. The discovery phase included 63 patients (UPPE: 30 patients; nonpurulent CPPE: 13 patients; empyema: 20 patients) and was based on a multiplex immunobead-based assay. The validation phase included 200 patients (transudates: 40 patients; other exudates: 31 patients; MPE: 49 patients; UPPE: 40 patients (n = 22 from the discovery phase and 18 new patients); nonpurulent CPPE: 18 patients (n = 8 from the discovery phase and 10 new patients); empyema: 22 patients (n = 17 from the discovery phase and 5 new patients) and was based on ELISA assays.

### Diagnostic criteria

Pleural effusions are classically divided into transudates and exudates based on Light's criteria^4^. A pleural effusion is categorized as MPE if malignant cells are demonstrated in pleural fluid or pleural biopsy^3^. PPE refers to a pleural fluid collection resulting from bacterial pneumonia, lung abscess, or bronchiectasis and is further classified as UPPE, nonpurulent CPPE, or empyema. Nonpurulent CPPE is defined as the presence of PPE with one of the following additional criteria: (1) pH < 7.2; (2) glucose < 60 mg/dl; (3) LDH > 1000 U/l; or (4) bacteria found on Gram’s stain or culture. Frank pus is termed empyema. Otherwise, UPPE is considered to exist when the patient’s exudative pleural fluid meets only one or none of the above criteria. After collection, the effusions were centrifuged at 3000 rcf for 10 min. Acellular supernatants were collected and stored at − 80 °C until use in the experimentsy^11^.

### Multiplex analysis of 40 cytokines and chemokines

The pleural levels of cytokines and chemokines were determined through the use of Bio-Plex Pro™ Assays (Bio-Rad Laboratories, CA, USA) in the multiplex suspension array system. The Bio-Plex Pro Human Chemokine Assay (171-AK99MR2) measures 40 targets: B cell-attracting chemokine 1 (BCA-1), cutaneous T-cell-attracting chemokine (CTACK), epithelial-derived neutrophil-activating peptide 78 (ENA-78), eosinophil chemotactic protein 1 (eotaxin-1), eosinophil chemotactic protein 2 (eotaxin-2), eosinophil chemotactic protein 3 (eotaxin-3), fractalkine, granulocyte chemotactic protein 2 (GCP-2), growth-regulated oncogene-α (Groα), growth-regulated oncogene-β (Groβ), granulocyte–macrophage colony-stimulating factor (GM-CSF), interferon γ (IFN-γ), IFN-γ-induced protein 10 (IP-10), interferon-inducible T-cell alpha chemoattractant (I-TAC), I-309, macrophage migration inhibitory factor (MIF), monocyte chemoattractant protein (MCP)-1, MCP-2, MCP-3, MCP-4, macrophage-derived chemokine (MDC), monokine induced by IFN-γ (MIG), macrophage inflammatory protein (MIP)-1α, MIP-3α, MIP-3β, MIP-1δ, myeloid progenitor inhibitory factor 1 (MPIF-1), interleukin (IL)-1β, IL-2, IL-4, IL-6, IL-8, IL-10, IL-16, stromal cell-derived factor 1 (SDF1), small inducible cytokine B16 (SCYB16), thymus-expressed chemokine (TECK), thymus- and activation-regulated chemokine (TARC), and tumor necrosis factor (TNF)-α. The assays were performed in 96-well microplates according to the protocol provided by Bio-Rad Laboratories.

### Enzyme-linked immunosorbent assay

Commercial sandwich ELISA kits (R&D Systems, MN, USA) were used to measure the levels of macrophage migration inhibitory factor (MIF), interleukin 1β (IL-1β), epithelial neutrophil-activating protein 78 (ENA-78), macrophage inflammatory protein-3 (MIP-3α), growth-regulated oncogene β (Groβ), and growth-regulated oncogene α (Groα) in pleural effusions. The assays were performed according to the manufacturer’s guidelines.

### Statistical analysis

In the multiplex immunobead-based assay, among the 40 cytokines we screened as the following strategy because of the limited samples (Table 2), (1) the fold changes of fluorescence intensities were upper 1.3 and lower 0.76 between CPPE and UPPE, (2) ten cytokines (ENA-78, IL-1β, Groβ, IL-8, MIP-3α, MIF, fractalkine, MCP3, MIP-1α, and Groα) were increased, (3) two cytokines (CTACK and SDF1) were decreased, (4) twelve cytokines were selected and examined the statistical significance between CPPE and UPPE using Mann–Whitney U test. 5. Bonferroni-adjusted p values < 0.05/24 (12 cytokines and 2 comparisons) = 0.0021 indicated significance.

The pleural levels of target proteins in ELISAs were showed as mean, median, and 25th–75th percentiles. Statistical analysis was done using the post hoc Turkey test for comparison the six cytokines among six different subgroups of patients. Bonferroni-adjusted p values were calculated as a α error 0.05 divided by numbers of cytokines and comparisons on each table.

ROC curves were generated by plotting sensitivity versus 1-specificity. The area under the curve (AUC) was reported with 95% confidence intervals as an estimate of diagnostic usefulness. The optimal cutoff value of each cytokine level was determined as the sum of its maximum sensitivity and specificity. Univariate logistic regression was performed to analyze the association between pleural biomarkers and the presence of CPPE. Unadjusted ORs were calculated as an estimate of risk. Spearman correlations were calculated to measure the associations among the candidate protein markers for PPE patients. All statistical tests were performed using SPSS software version 20 (SPSS Inc., Chicago, IL, USA).

## Supplementary Information

Supplementary Information.
