# Supplementary material for: Pleural cytokines MIF and MIP-3α as novel biomarkers for complicated parapneumonic effusions and empyema
Source: Sci Rep. 2021 Jan 19;11:1763. doi: 10.1038/s41598-021-81053-6 (PMC7815762; doi:10.1038/s41598-021-81053-6)
Supplement: Supplementary file 1 — Supplementary Information. [file 41598_2021_81053_MOESM1_ESM.docx]

**Supplementary Information**

**Pleural cytokines MIF and MIP-3α as novel biomarkers for complicated parapneumonic effusions and empyema**

Chia-Yu Yang^1,2,3^, Yu-Hsuan Kuo^1,4^, Min Chen^1,4^, Chih-Liang Wang^5,6^, Li-Jane Shih^7^, Yu-Ching Liu^4^, Pei-Chun Hsueh^4^, Yi-Hsuan Lai^4^, Chi-Ming Chu^8^, Chih-Ching Wu^2,3,9^, Kuo-An Wu^10,11^

1 Department of Microbiology and Immunology, College of Medicine, Chang Gung University, Taoyuan, Taiwan

2 Department of Otolaryngology-Head & Neck Surgery, Chang Gung Memorial Hospital, Taoyuan, Taiwan

3 Molecular Medicine Research Center, Chang Gung University, Taoyuan, Taiwan

4 Graduate Institute of Biomedical Sciences, College of Medicine, Chang Gung University, Taoyuan, Taiwan

5 School of Medicine, College of Medicine, Chang Gung University, Taoyuan, Taiwan

6 Division of Pulmonary Oncology and Interventional Bronchoscopy, Department of Thoracic Medicine, Chang Gung Memorial Hospital, Taoyuan, Taiwan

7 Department of Medical Laboratory, Taoyuan Armed Forces General Hospital, Taoyuan, Taiwan

8 Division of Biomedical Statistics and Informatics, School of Public Health, National Defense Medical Center, Taipei, Taiwan

9 Department of Medical Biotechnology and Laboratory Science, College of Medicine, Chang Gung University, Taoyuan, Taiwan

10 Department of Internal Medicine, Taoyuan Armed Forces General Hospital, Taoyuan, Taiwan

11 School of Medicine, Fu-Jen Catholic University, New Taipei City, Taiwan

**
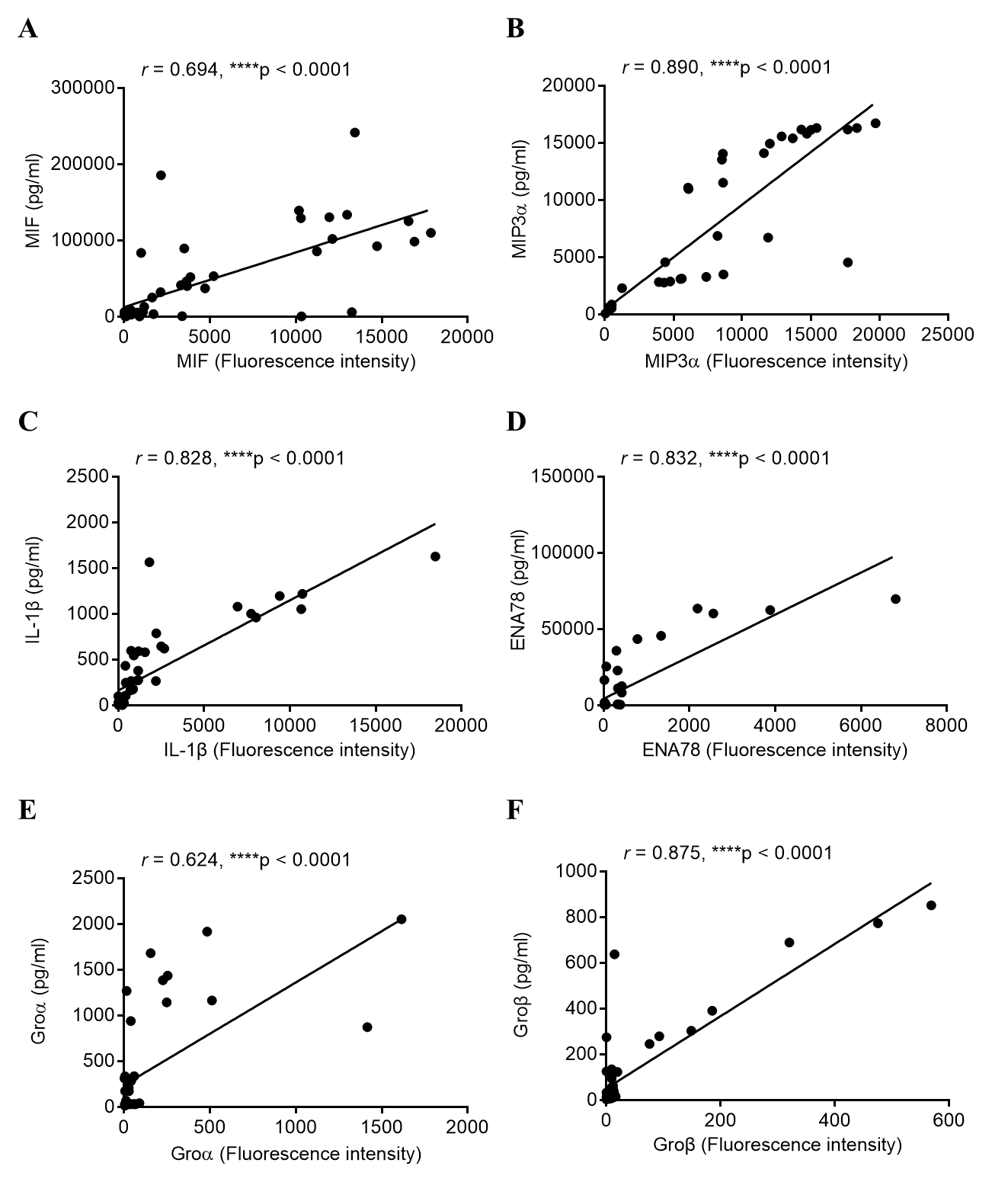
**

**Supplementary Figure 1. Correlation between multiplex immunoassay and ELISA results.** Correlation of pleural fluid MIF (A), MIP-3α (B), IL-1β (C), ENA78 (D), Groα (E), and Groβ (F) levels measured on different platforms. Statistically significant correlations were determined using the Pearson correlation. The Pearson r-value and p-value are indicated in the graph. Bonferroni-adjusted p-values < 0.05/6 (6 cytokines) = 0.0083 indicated significance.

**
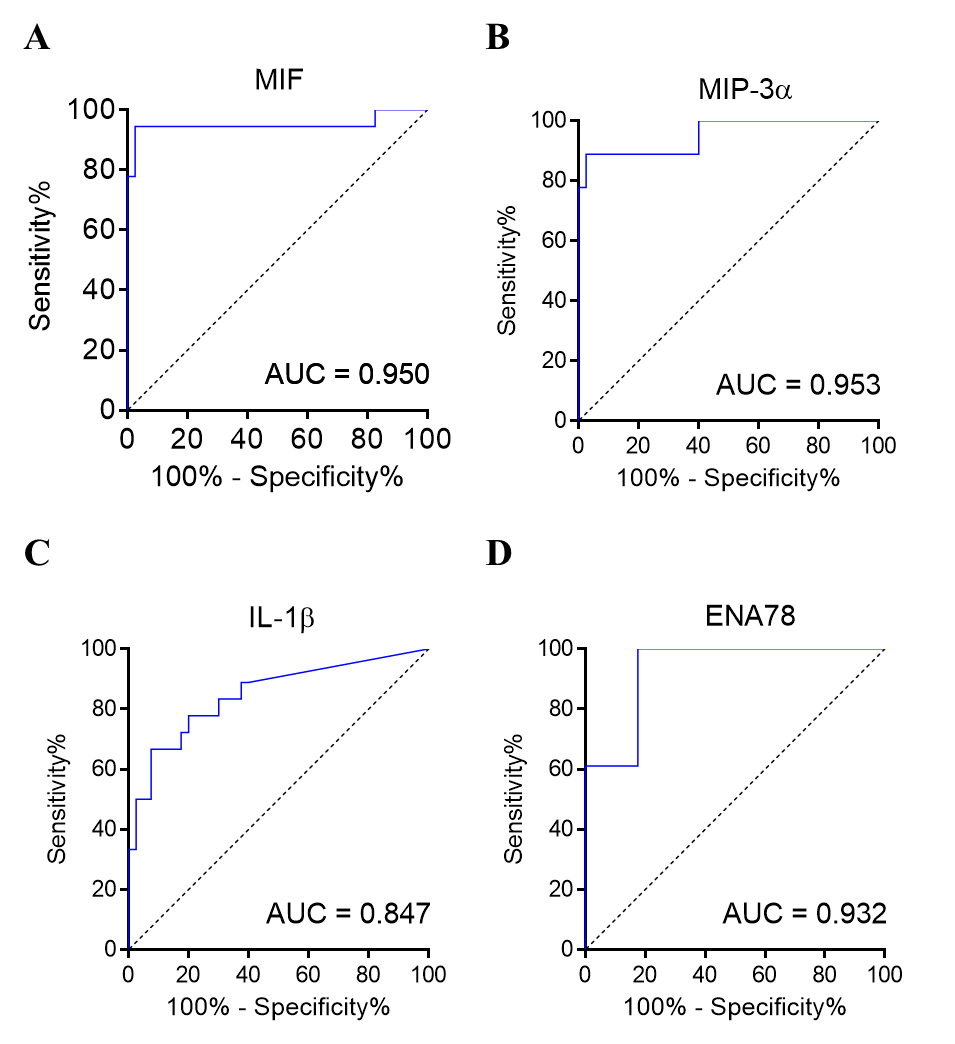
**

**Supplementary Figure 2. ROC curves of pleural fluid cytokines for distinguishing CPPE from UPPE.** ROC curves of pleural fluid MIF (A), MIP-3α (B), IL-1β (C), and ENA78 (D) were plotted with GraphPad Prism software.

**Supplementary Table 1. Biological functions of the 40 proteins measured in the Bio-Plex assay**

| **Protein** | **Biological process^a^** | **Protein** | **Biological process^a^** |
| --- | --- | --- | --- |
| **Cytokines^b^** |  |  |  |
| IL-1β | inflammation | IL-10 | inflammation, immune response |
| IL-2 | adaptive immunity | IL-16 | lymphocyte chemotaxis, T-cell immune response |
| IL-4 | humoral immune response | IFN-r | immune response, antiviral defense, growth regulation |
| IL-6 | humoral immune response, acute phase response | MPIF-1 | inflammation, innate immunity |
| IL-8 | neutrophil, basophil and T-cell chemotaxis, inflammation, angiogenesis | TNF-α | inflammation, innate immunity |
| **Chemokines^b^** |  |  |  |
| BCA-1/CXCL13 | chemotaxis, inflammation, immune response | MCP-2/CCL8 | monocyte and neutrophil chemotaxis, inflammation |
| CTACK /CCL27 | memory T-cell chemotaxis, immune response | MCP-3/CCL7 | monocyte and eosinophil chemotaxis, inflammation |
| ENA-78/CXCL5 | chemotaxis, inflammation, immune response | MCP-4/CCL13 | monocyte and neutrophil chemotaxis, inflammation |
| Eotaxin-1/CCL11 | eosinophil chemotaxis, inflammation | MDC/CCL22 | chemotaxis, inflammation, immune response |
| Eotaxin-2/CCL24 | chemotaxis, inflammation, immune response | MIG/CXCL9 | activated T-cell chemotaxis, inflammation |
| Eotaxin-3/CCL26 | chemotaxis, inflammation, immune response | MIP-1α/CCL3 | chemotaxis, inflammation, immune response |
| Fractalkine/CX3CL1 | chemotaxis, defense response, immune response | MIP-3α/CCL20 | monocyte and neutrophil chemotaxis, inflammation |
| GCP-2/CXCL6 | chemotaxis, inflammation, immune response | MIP-3β/CCL19 | monocyte chemotaxis, inflammation, immune response |
| Gro-α/CXCL1 | neutrophil chemotaxis, inflammation | MIP-1δ/CCL15 | chemotaxis, inflammation |
| Gro-β/CXCL2 | neutrophil chemotaxis, inflammation | MIF | chemotaxis, inflammation, immune response |
| IP-10/CXCL10 | monocyte and T-cell chemotaxis, inflammation | SCYB16/CXCL16 | chemotaxis |
| I-TAC/CXCL11 | chemotaxis, inflammation, immune response | TECK/CCL25 | chemotaxis, inflammation, immune response |
| I-309/CCL1 | chemotaxis, inflammation | 6Ckine/CCL21 | chemotaxis, inflammation, immune response |
| MCP-1/CCL2 | monocyte and basophil chemotaxis, inflammation |  |  |
| **Growth factors^b^** |  |  |  |
| GM-CSF | immune response, angiogenesis | SDF-1/CXCL12 | monocyte and T-cell chemotaxis, angiogenesis |
| **Other^b^** |  |  |  |
| TARC/CCL17 | immune response regulation |  |  |

**Supplementary Table 2. Spearman correlations of six proteins in PPE pleural fluid with clinical parameters.**

|  | MIF | ENA78 | MIP3α | Groβ | Groα | IL1β | Glucose | LDH | pH |
| --- | --- | --- | --- | --- | --- | --- | --- | --- | --- |
| MIF | 1.000 | 0.739** | 0.804** | 0.537** | 0.292** | 0.714** | -0.595** | 0.744** | -0.690** |
| ENA78 | – | 1.000 | 0.718** | 0.734** | 0.542** | 0.672** | -0.634** | 0.666** | -0.676** |
| MIP3α | – | – | 1.000 | 0.569** | 0.221* | 0.636** | -0.505** | 0.703** | -0.622** |
| Groβ | – | – | – | 1.000 | 0.590** | 0.492** | -0.432** | 0.505** | -0.501** |
| Groα | – | – | – | – | 1.000 | 0.413** | -0.324** | 0.316** | -0.237* |
| IL1β | – | – | – | – | – | 1.000 | -0.708** | 0.676** | -0.742** |
| Glucose | – | – | – | – | – | – | 1.000 | -0.593** | 0.710** |
| LDH | – | – | – | – | – | – | – | 1.000 | -0.744** |
| pH | – | – | – | – | – | – | – | – | 1.000 |

* P < 0.05, ** P < 0.01
